# Strongyloides Stercoralis Infection: A Rare Cause of Acute Abdomen

**DOI:** 10.7759/cureus.11470

**Published:** 2020-11-13

**Authors:** Ashley R Gao, Abhishek Matta

**Affiliations:** 1 Internal Medicine, University of North Dakota, Fargo, USA

**Keywords:** strongyloides stercoralis, strongyloidiasis, acute abdomen, stool ova and parasite exam

## Abstract

A 30-year-old female presented to the emergency department with severe recurrent abdominal pain, nausea, vomiting and a 50-pound unintentional weight loss within the past few years. After emigrating from Liberia to the United States in 2005, the patient was evaluated for similar complaints in the past and underwent exploratory laparotomies and partial small bowel resection with no significant improvement in symptoms. Computerized tomography (CT) of the abdomen showed intestinal inflammation and mesenteric edema. Small intestinal enteroscopy was unremarkable. Small intestinal biopsy showed larval round worms in intestinal crypts. Stool ova and parasite exam revealed larval forms of Strongyloides stercoralis. She was treated with ivermectin 200 mcg/kg daily for two days and recommended to return in four weeks to repeat stool exam to ensure complete parasite clearance.

## Introduction

Endemic to tropical regions with inadequate access to sanitation, Strongyloidiasis is caused by infection with Strongyloides stercoralis, a soil-transmitted intestinal nematode. After penetrating the skin, infectious filariform larvae travel to the lungs via the venous circulation, ascend the tracheobronchial tree, and then are swallowed into the gastrointestinal tract [1]. Roughly 50% of infections are subclinical or asymptomatic; however, chronic strongyloidiasis can be associated with abdominal pain, anorexia, nausea, vomiting, diarrhea, or constipation.

Strongyloidiasis can be diagnosed definitively with a stool ova and parasite exam showing the presence of rhabditiform larvae. The diagnosis is further supported by eosinophilia and thorough history-taking, with special attention to travel history [1]. Although common in endemic regions, it can also be seen in developed countries due to migrating populations from endemic regions. A high degree of clinical suspicion should be maintained to avoid performing unnecessary surgeries, as seen in this case.

## Case presentation

A 30-year-old female presented to the emergency department with severe recurrent abdominal pain, nausea, vomiting and a 50-pound unintentional weight loss over the last few years. She underwent exploratory laparotomies and partial small bowel resection for similar complaints in the past. She denied any fevers, night sweats, or shortness of breath and reported no changes to appetite or bowel movements. 

The patient emigrated from Liberia to the United States 15 years ago but has not traveled out of the country recently. Her first abdominal surgery was more than 10 years ago for suspected bowel obstruction. In 2019, she had two hospital admissions for similar abdominal issues suspected to be related to bowel obstruction, which were managed conservatively. Most recently in March 2020, she had another exploratory laparotomy for bowel obstruction and underwent adhesiolysis. 

During this admission, vital signs were stable upon initial evaluation. The physical exam was remarkable for abdominal distension and multiple abdominal surgical scars. Laboratory studies are summarized in Table 1. Serum inflammatory markers including white blood count, eosinophil count, erythrocyte sedimentation rate, and C-reactive protein were normal but stool markers such as fecal lactoferrin and calprotectin were elevated. 

**Table 1:** Laboratory studies done throughout hospitalization

Test	Value	Reference Range
WBC	8.6	4.0-11.0 K/µL
Eosinophils Absolute	0.1	0.0-0.7 K/µL
ESR	14	0-20 mm/hr
CRP	6.7	0-8.0 mg/L
Fecal calprotectin	787.2	<50.0 mcg/g
Fecal lactoferrin	Positive	Negative
QuantiFERON	Positive	Negative
Saccharomyces cerevisiae IgG	29.6	≦20 Negative 20.1- 29.9 Equivocal ≧30 Positive
Saccharomyces cerevisiae IgA	<20.0	≦20 Negative 20.1- 24.9 Equivocal ≧25 Positive

CT abdomen and pelvis obtained at the time of admission showed mesenteric edema, diffuse small bowel thickening, and a bowel suture in the right mid-abdomen consistent with prior small bowel resection. Subsequent small bowel enteroscopy showed duodenal and jejunal inflammation with edema, erosions, erythema, friability, and aphthous ulcerations. Colonoscopy revealed aphtha in the transverse and ascending colon, the appearance concerning for colitis. Biopsies of the small intestine and colon were taken at this time. 

**Figure 1:**
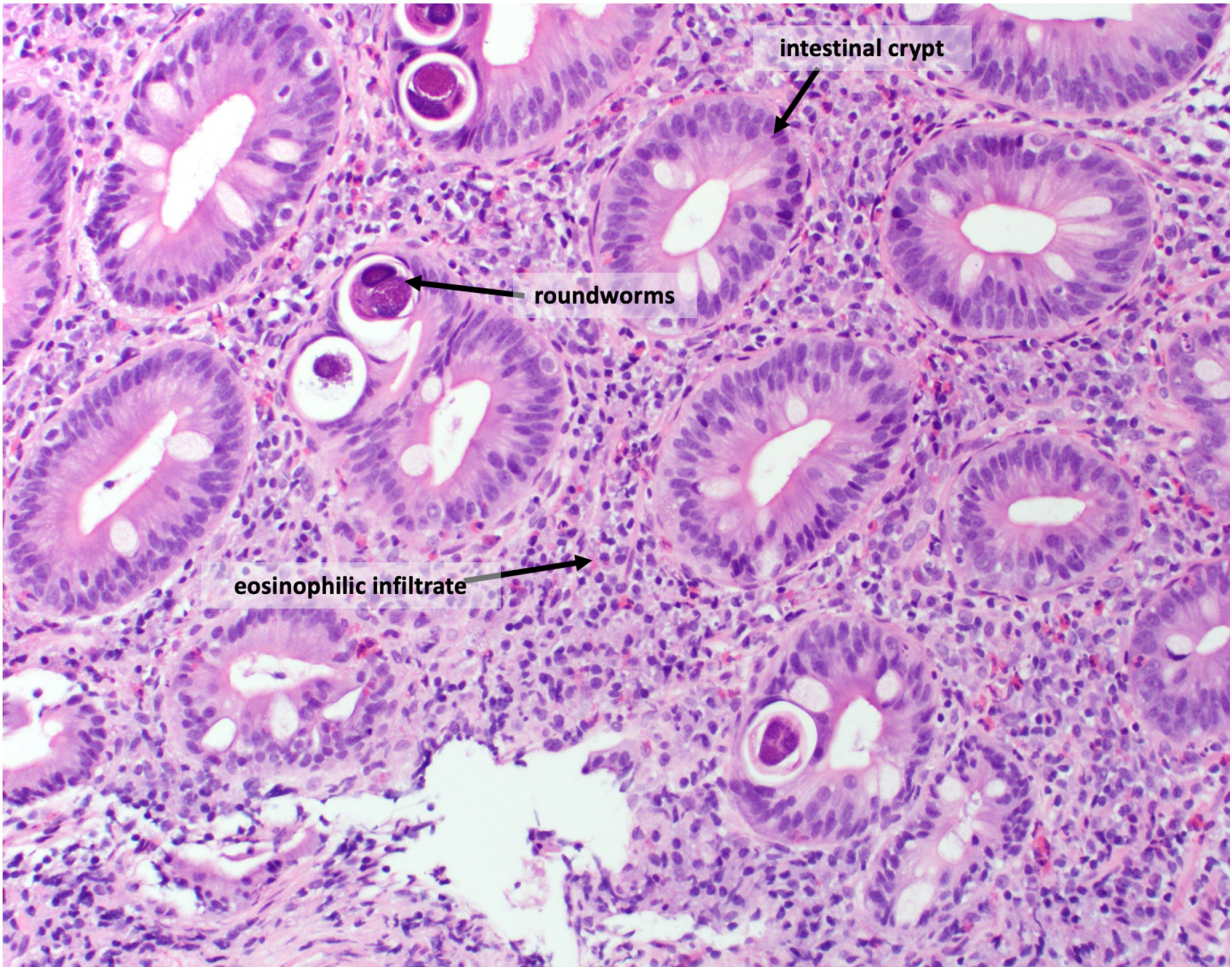
Strongyloides stercoralis larvae in small intestine tissue specimens, stained with H&E H&E, hematoxylin and eosin

**Figure 2:**
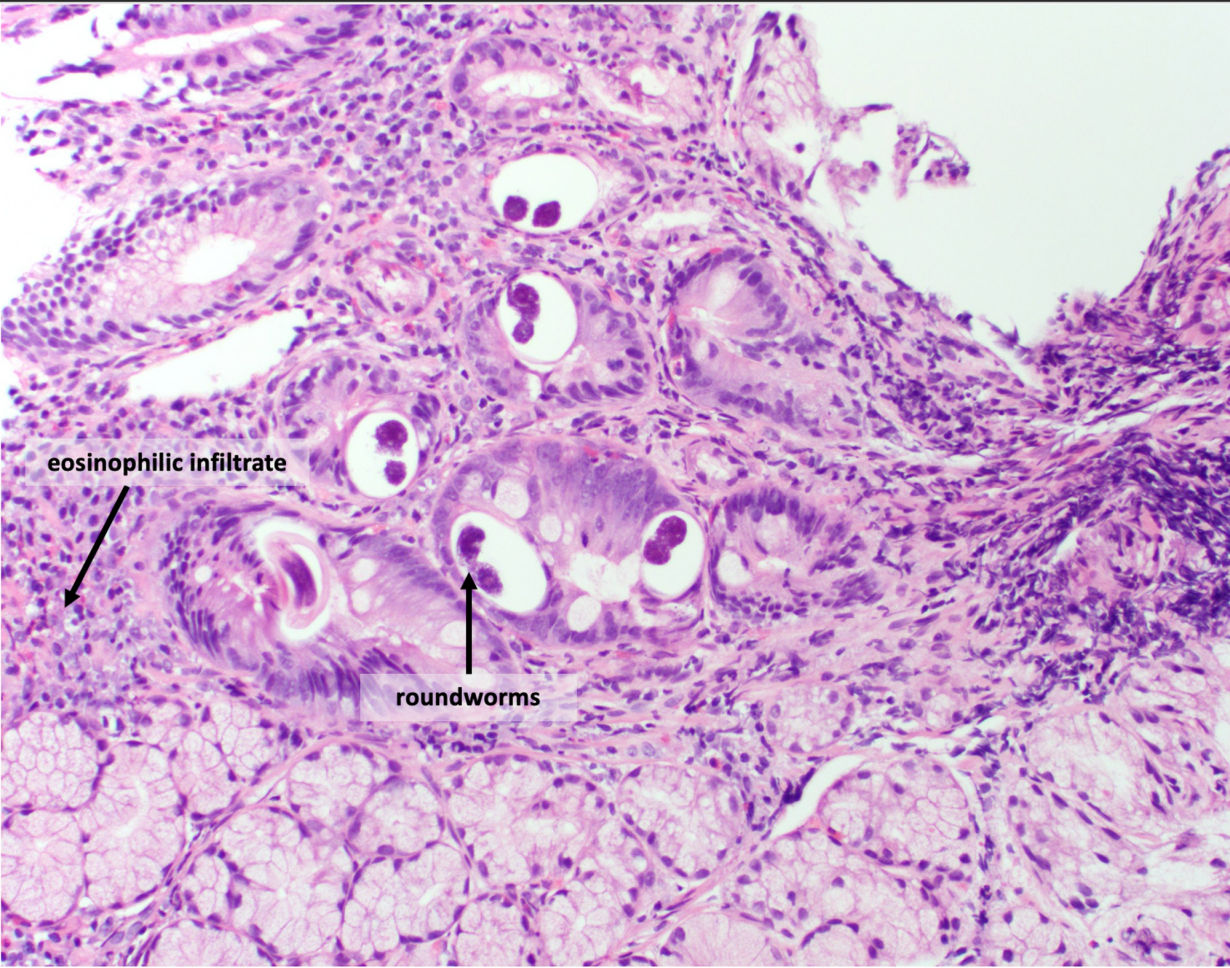
Strongyloides stercoralis larvae in small intestine tissue specimens, stained with H&E H&E, hematoxylin and eosin

Biopsies came back positive for duodenal and jejunal lymphoplasmacytic, neutrophilic, and eosinophilic inflammation of the lamina propria. Discrete parasitic forms were present in the epithelium and lamina propria, most suggestive of roundworms (Figures 1, 2). The specimens were negative for increased intraepithelial lymphocytosis, granulomata, and dysplasia. Bowel samples were also negative for tuberculosis on the acid-fast smear. 

**Figure 3:**
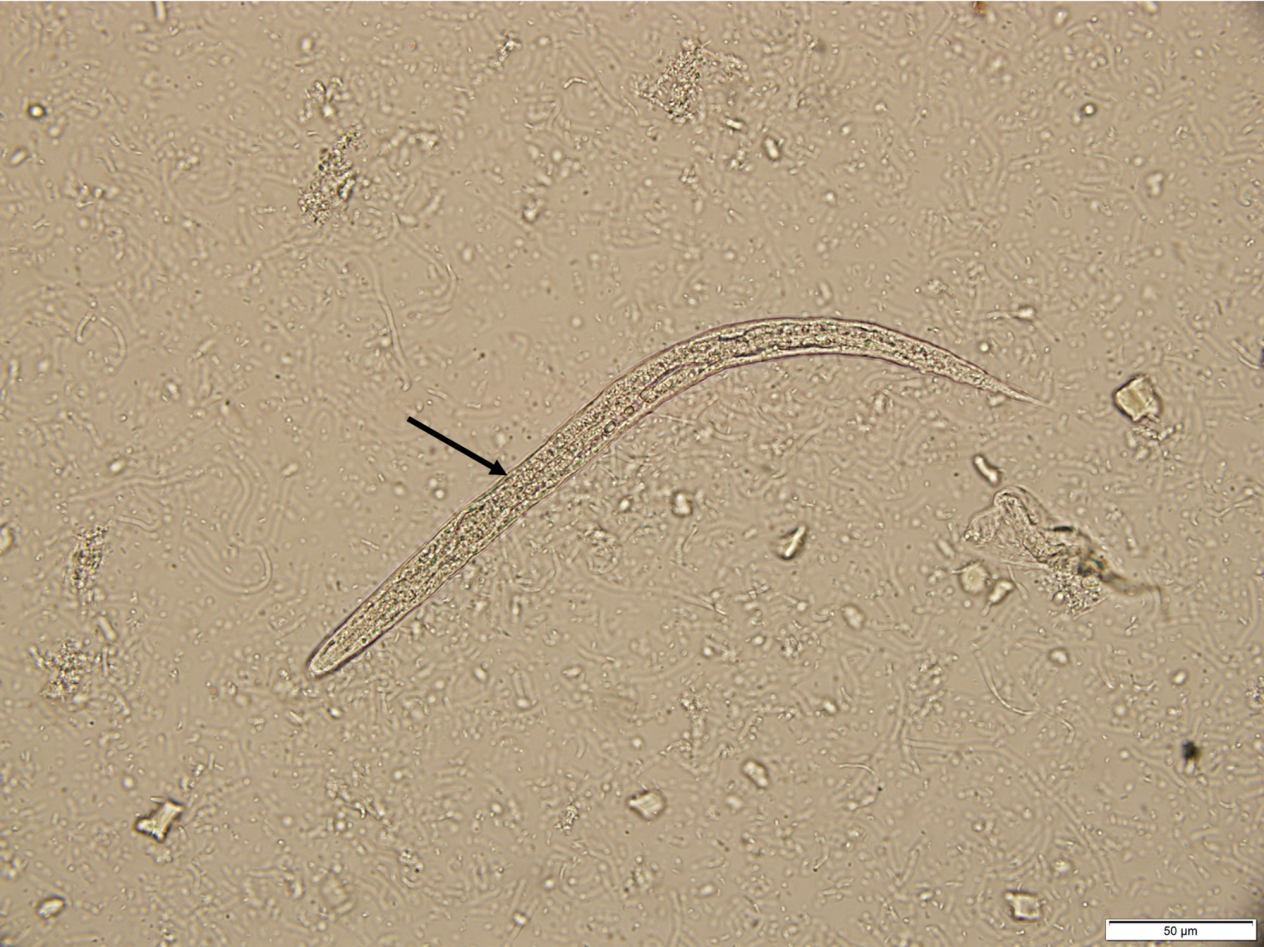
Stool wet mount shows Strongyloides stercoralis rhabditiform larvae

**Figure 4:**
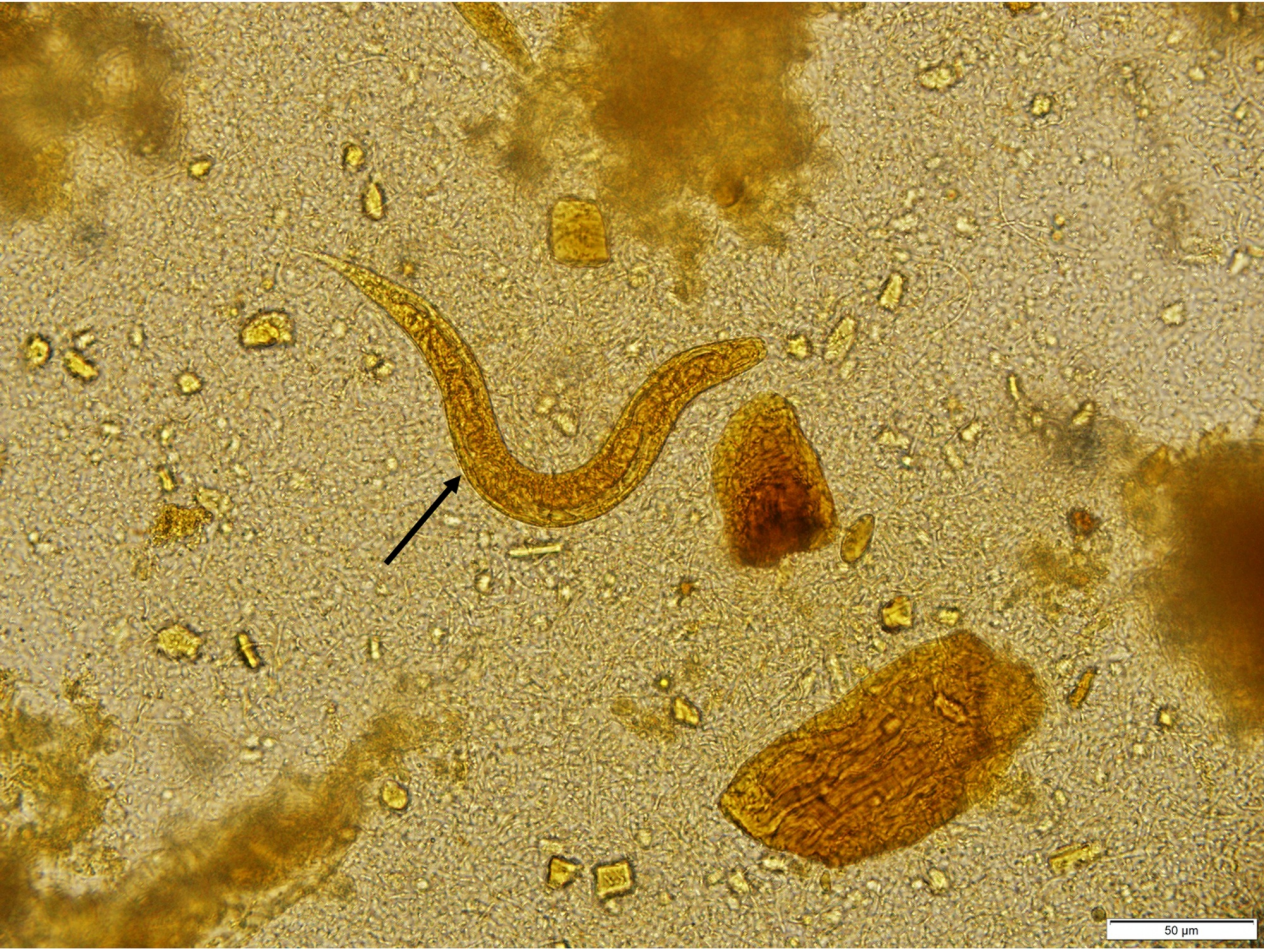
Lugol iodine stain shows Strongyloides stercoralis rhabditiform larvae in stool

Stool ova and parasite exam subsequently detected Strongyloides stercoralis rhabditiform larvae (Figures 3, 4). The patient was treated with ivermectin 200 mcg/kg daily for two days and recommended to return in four weeks to repeat stool exam to ensure complete parasite clearance. 

## Discussion

Strongyloidiasis, despite its low prevalence in the United States, should still be suspected in patients coming from endemic regions who present with acute abdomen [1,2]. A high degree of clinical suspicion should be maintained to avoid performing unnecessary surgeries. Roughly 50% of infections are subclinical or asymptomatic; however, untreated strongyloidiasis can be associated with recurrent abdominal pain, anorexia, nausea, vomiting, diarrhea, or constipation [2,3]. While gastrointestinal symptoms are most common, cutaneous larva currens, in which pruritic linear lesions appear due to larvae migration, is considered pathognomonic [4].

Characterized by a unique life cycle, larvae may be passed in the stool or remain in the host, causing autoinfection for many decades [5]. When the host’s bare skin comes in contact with contaminated soil, infectious filariform larvae penetrate the skin and travel through the circulatory system to the lungs. The filariform larvae then penetrate through alveoli, enter the trachea and pharynx, before being swallowed into the gastrointestinal system. After molting twice in the small intestine, they reach adulthood. The adult female worms deposit eggs in the intestinal epithelium, which then hatch into rhabditiform larvae. The rhabditiform larvae can be passed in stool or cause autoinfection. To establish autoinfection, the rhabditiform larvae develop into the infectious filariform larvae, which undergo internal autoinfection (by penetrating the intestinal mucosa) or external autoinfection (by penetrating the perianal skin) [4].

In immunosuppressed individuals, autoinfection leads to a hyperinfection syndrome, resulting in over-proliferation of larvae with dissemination to organs outside of the usual migration pattern [1]. The most common extraintestinal manifestation in the immunosuppressed is respiratory [6]. Pulmonary migration of larvae presents as Löffler syndrome (eosinophilic pneumonia) in which cough, wheezing, and shortness of breath are common complaints [1,3,6]. Multi-organ dissemination results in secondary bacteremia as the migrating larvae damage intestinal tissue causing leakage of gut flora, with mortality rates as high as 87% [5,6].

Clinically, stool ova and parasite exam should be utilized when signs of acute abdomen are observed in patients with a history of travel to endemic regions. Currently, the standard treatment is ivermectin 200 mcg/kg daily for two days, and patients should be recommended to return in two to four weeks for repeat stool exam to ensure complete parasite clearance [1]. 

Our patient probably had the infection for a long time given her multiple hospital admissions and emergency room visits for identical symptoms. Even though she had multiple abdominal surgeries and even a partial bowel resection, no etiology could be identified. The mesenteric edema and lymphadenopathy seen on CT during this hospital stay were the result of the inflammatory response triggered by the parasite in the intestinal crypts and raised suspicion that prompted further investigation. Our differential diagnosis at the time included inflammatory bowel disease. Biopsy of the small intestine showed the presence of parasites in the intestinal crypts. The diagnosis was confirmed with a stool ova and parasite exam showing the presence of Strongyloides stercoralis rhabditiform larvae and additionally supported by thorough history-taking with special attention to travel history [1].

## Conclusions

Strongyloidiasis has low prevalence in the United States but should still be suspected in patients traveling from endemic regions who present with acute abdomen. Common symptoms of untreated strongyloidiasis include recurrent abdominal pain, anorexia, nausea, vomiting, diarrhea, or constipation. Diagnosis is made with stool ova and parasite exam showing the presence of Strongyloides stercoralis larvae. First-line therapy for Strongyloidiasis is ivermectin 200 mcg/kg daily for two days and patients are recommended to return in two to four weeks to repeat stool exam to ensure complete parasite clearance.
